# Experiences of Young People and Their Caregivers of Using Technology to Manage Type 1 Diabetes Mellitus: Systematic Literature Review and Narrative Synthesis

**DOI:** 10.2196/20973

**Published:** 2021-02-02

**Authors:** Nicola Brew-Sam, Madhur Chhabra, Anne Parkinson, Kristal Hannan, Ellen Brown, Lachlan Pedley, Karen Brown, Kristine Wright, Elizabeth Pedley, Christopher J Nolan, Christine Phillips, Hanna Suominen, Antonio Tricoli, Jane Desborough

**Affiliations:** 1 Department of Health Services Research and Policy Research School of Population Health, College of Health and Medicine Australian National University Canberra Australia; 2 Canberra Health Services Canberra Australia; 3 ANU Medical School College of Health and Medicine Australian National University Canberra Australia; 4 The John Curtin School of Medical Research College of Health and Medicine Australian National University Canberra Australia; 5 School of Computing College of Engineering and Computer Science Australian National University Canberra Australia; 6 Department of Computing University of Turku Turku Finland; 7 Data61 Commonwealth Scientific and Industrial Research Organisation Canberra Australia; 8 Nanotechnology Research Lab Research School of Chemistry, College of Science Australian National University Canberra Australia

**Keywords:** type 1 diabetes mellitus, diabetes, children, adolescents, technology, self-management, experiences, perspectives, systematic review

## Abstract

**Background:**

In the last decade, diabetes management has begun to transition to technology-based care, with young people being the focus of many technological advances. Yet, detailed insights into the experiences of young people and their caregivers of using technology to manage type 1 diabetes mellitus are lacking.

**Objective:**

The objective of our study was to describe the breadth of experiences and perspectives on diabetes technology use among children and adolescents with type 1 diabetes mellitus and their caregivers.

**Methods:**

This systematic literature review used integrated thematic analysis to guide a narrative synthesis of the included studies. We analyzed the perspectives and experiences of young people with type 1 diabetes mellitus and their caregivers reported in qualitative studies, quantitative descriptive studies, and studies with a mixed methods design.

**Results:**

Seventeen articles met the inclusion criteria, and they included studies on insulin pump, glucose sensors, and remote monitoring systems. The following eight themes were derived from the analysis: (1) expectations of the technology prior to use, (2) perceived impact on sleep and overnight experiences, (3) experiences with alarms, (4) impact on independence and relationships, (5) perceived usage impact on blood glucose control, (6) device design and features, (7) financial cost, and (8) user satisfaction. While many advantages of using diabetes technology were reported, several challenges for its use were also reported, such as cost, the size and visibility of devices, and the intrusiveness of alarms, which drew attention to the fact that the user had type 1 diabetes mellitus. Continued use of diabetes technology was underpinned by its benefits outweighing its challenges, especially among younger people.

**Conclusions:**

Diabetes technologies have improved the quality of life of many young people with type 1 diabetes mellitus and their caregivers. Future design needs to consider the impact of these technologies on relationships between young people and their caregivers, and the impact of device features and characteristics such as size, ease of use, and cost.

## Introduction

### Background

Type 1 diabetes mellitus (T1DM) is a chronic autoimmune disease that results in elevated blood glucose levels due to destruction of insulin-producing pancreatic islet β cells [1]. It is frequently diagnosed among children and adolescents, with the peak age group of diagnosis being 10 to 19 years [2,3]. Globally, the prevalence of T1DM among children and adolescents equates to over 1 million people currently affected [4]. Continuous glucose monitoring (CGM) has been found to have a positive impact on young people’s health-related quality of life [5,6]; therefore, technology-supported care approaches specifically for children and adolescents continue to be developed and improved [7]. Further adaptation of diabetes technology for use by young people and their caregivers can optimize diabetes management and outcomes from an early age. Insight into the experiences of young people and their caregivers of using devices to manage T1DM is essential to guide device developers and health care professionals to optimize the use and function of these technologies [8,9].

### Diabetes Management in Youth

Disease management at an early age requires interdisciplinary care coordination between the child, the parents/family, the health care professional team [10], and others involved in care, such as teachers [11]. The diagnosis of diabetes at a young age is frequently accompanied by psychological stress in both the child or adolescent and parents related to the disease management demands (24 hours a day, 7 days a week), including the integration of complex treatment regimens [12] and fear of the consequences of poor blood glucose control, particularly hypoglycemia [13,14]. For adolescents, diabetes management can be a major challenge as a consequence of growing independence from parents, increasing complexity of daily activities (eg, managing diabetes technology), the added psychological demands associated with this age including peer pressure [11], and the pubertal physiological changes in the body.

### Technology for Diabetes Management

To achieve optimal blood glucose control, adolescents with T1DM have to manage the following three key components: (1) glucose monitoring, (2) insulin delivery, and (3) means of communication between (1) and (2). Exogenous insulin administration into subcutaneous tissues by insulin injection or infusion by pump is informed by measurement of either blood glucose or subcutaneous interstitial fluid glucose. Such treatment is necessary to avoid short-term complications (eg, hypoglycemic events and diabetic ketoacidosis) and long-term complications (eg, diabetic retinopathy and nephropathy) [1,15]. For glucose monitoring, the choices include finger stick blood sampling for self-monitoring of blood glucose (SMBG) and/or continuous subcutaneous interstitial fluid glucose measurement with real-time access using CGM systems and/or intermittent access using flash glucose monitoring (FGM) systems. The choices for insulin delivery are multiple dose injections or continuous subcutaneous insulin infusion (CSII) by pump [16]. All combinations of glucose monitoring and insulin delivery devices are used in current practice [17]. Until recently, there were no direct electronic means of communication between the glucose monitoring and insulin delivery systems, such that a young person with diabetes or a parent/caregiver would need to make all decisions. New technology, however, has brought new means of communication between glucose sensing devices, people with diabetes, and insulin delivery systems [16]. Safety features, such as “suspend before low,” and glucose sensing-insulin infusion closed loop (CL) systems, can now be used. Hybrid closed loop (HCL) systems, in which the operating person provides some information into the otherwise CL system, such as carbohydrate intake amount that triggers an insulin bolus, are now commercially available. Table 1 provides a comprehensive technology overview [18-25].

Previous reviews on diabetes technology have mostly focused on the effectiveness or efficacy of the technology in adult populations [26-28], with some also including youth [29]. While various studies have focused on experiences with diabetes technology and particularly experiences with technology in young people with T1DM, reviews of such study findings are still lacking. Therefore, this systematic integrative review aimed to describe the breadth of experiences and perspectives on diabetes technology use among adolescents with T1DM and their caregivers.

**Table 1:** Explanations of diabetes technology abbreviations and systems.

Technology	Acronym	Explanation
Real-time continuous glucose monitoring	RT-CGM	This device has a glucose sensor that measures the wearer’s levels of glucose in the interstitial fluid. A signal transmits continuously via radio frequency to a receiver, where the user can see glucose levels in real-time intervals of a few minutes [18,19].
Continuous subcutaneous insulin infusion	CSII	This form of insulin therapy has been in use for some time. Short-acting insulin is provided through a pump. The dose is adjusted to meet the individual user’s insulin needs, established with experience over time [19].
Cell phone glucose monitoring	CPGM	This cell phone–based system transmits the user’s blood glucose levels to a host computer, which is monitored by a health care professional [20].
Flash glucose monitoring	FGM	This device has a sensor that monitors the user’s levels of glucose in interstitial fluid. The user physically swipes a reader device over the sensor to transmit a real-time glucose level and 8 hours of retrospective data, including a trend line [21,22].
Hybrid closed loop system	HCL	The system is a package comprised of an insulin pump and a CGM^a^ system. It can function in the following two different modes: “auto mode” (CL^b^) and “manual mode” (HCL^c^). In CL (auto mode), basal insulin delivery is automatically adjusted in response to CGM levels that are transmitted to the insulin pump. CL is sometimes also called “artificial pancreas” as it requires minimal input from the user. In HCL (manual mode), preprogrammed insulin doses are infused throughout the day, and users must manually deliver bolus doses at meal times and other times to correct blood glucose levels [23,24].
Multiple dose injection therapy	MDI	This system of insulin delivery has been in use for a long time. It involves subcutaneous injections of either long- or rapid-acting insulin. Long-acting insulin is usually injected once or twice daily and rapid-acting insulin is injected at meal times [25].
Sensor-augmented pump therapy	SAPT	This system combines CSII and CGM. The glucose sensor is introduced directly into the CSII, and as the name indicates, augments insulin pump therapy [19].

^a^continuous glucose monitoring.

^b^closed loop.

^c^hybrid closed loop.

## Methods

### Review Design

This systematic literature review was based on the design synthesis methods of the Evidence for Policy and Practice Information Centre (EPPI-Centre) [30] and the integrative review methodology described by Whittemore and Knafl [31]. Integrative reviews enable the synthesis of data from diverse sources (qualitative and quantitative) to provide a broad and holistic understanding of the subjective and objective elements of a topic, including context, processes, and outcomes [31]. Integrated thematic analysis of data guided a narrative synthesis of the results. Data from qualitative, quantitative, and mixed methods studies were included in this narrative synthesis. The review was registered with PROSPERO (registration number: CRD42019125351).

### Patient and Public Involvement

In the true spirit of patient and public involvement in research, our team included academics, clinicians, three young people with T1DM, and two of their parents. All team members have contributed to this review, including identifying appropriate search terms, assisting with data extraction and data analysis, and providing comments on various drafts of the manuscript.

### Search Strategy

We searched PubMed, CINAHL, MEDLINE, Scopus, ProQuest, and Web of Science (search in title/abstract). The search string included the following keywords: (“Type 1 diabetes” OR “insulin dependent diabetes mellitus” OR “juvenile diabetes”) AND (“self manage*” OR “self measur*” OR “self monitor*”) AND (adolescent OR children) AND experienc*. We did not use the term “technology” or a similar term in the search string because this limited the results considerably (a comparison was conducted). The reference lists of included studies were searched to include studies that did not appear in the database search. The Cochrane software Covidence [32] was used to assist in the systematic review process from screening to data extraction.

### Inclusion/Exclusion Criteria

Owing to the lack of age specification in many studies, we included studies with participants aged 12 to 25 years to ensure we captured adolescents, who were our primary interest. Studies that focused on parents’ or caregivers’ experiences of caring for a young person with T1DM were also included. We included peer-reviewed studies conducted in any country and in English language from 2009 to early 2019. We excluded randomized controlled trials (RCTs) owing to the integrative narrative scope of the review, which aimed to understand experiences rather than efficacy and effectiveness of technology. Other systematic reviews, conference abstracts, and grey literature were excluded.

### Screening and Quality Assessment

Selected studies were reviewed independently by two researchers, based first on the title and abstract and then on full-text review. Conflicts were resolved through discussion with a third independent reviewer. A full-text quality appraisal was performed independently by two reviewers using the Mixed Methods Appraisal Tool (MMAT) [33].

### Data Analysis

We combined the study findings in a thematic narrative synthesis. Differences by technologies (CGM, cell phone glucose monitoring [CPGM], FGM, HCL, CL, insulin pumps/bolus advisors, and sensor-augmented pump therapy [SAPT]) were identified within the narrative. Owing to the integrative narrative character of our review, we did not conduct a meta-analysis or report statistical results. This is in line with the narrative synthesis method used in previous systematic reviews [34-36]. We used the quality assessment of the respective studies/papers (MMAT) to ensure credibility of the papers.

## Results

### Data Extraction and Synthesis

**Figure 1:**
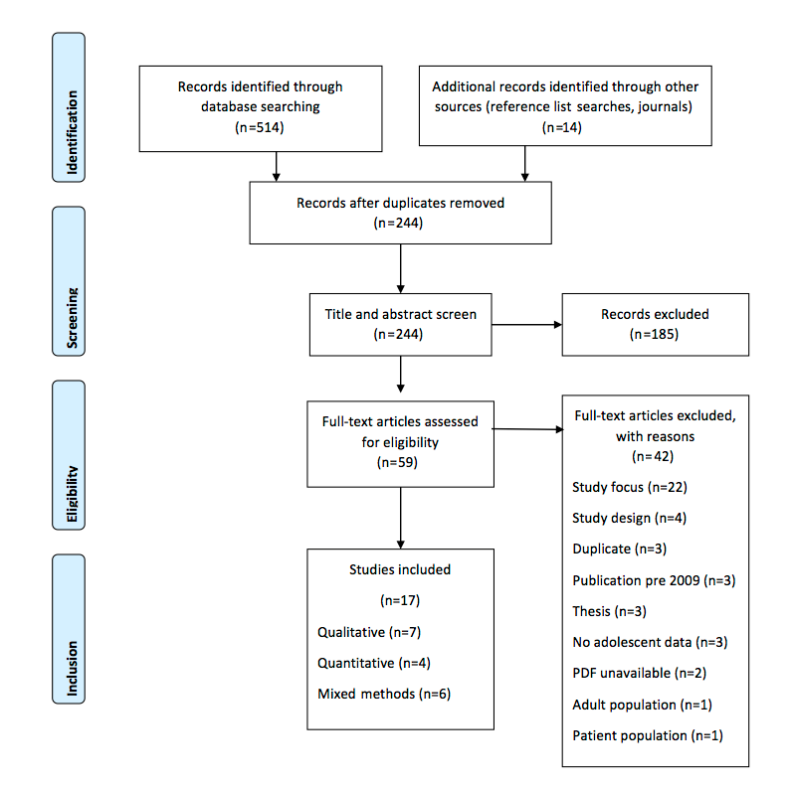
PRISMA (Preferred Reporting Items for Systematic Reviews and Meta-Analyses) flow diagram.

Of 528 identified references, 59 were selected for full-text review. A total of 17 studies were included. Of these, seven studies used qualitative research methods [37-43], four used quantitative methods [20,44-46], and six used mixed method designs [47-52], with only the quantitative component [50] or qualitative component [49,51] of three studies included (Figure 1).

Data were extracted to summarize study characteristics, including study descriptors, technology used, study aims, methods, main findings, and included themes (Multimedia Appendix 1). Data were coded into categories that were classified into eight themes following in-depth discussion and comparison. These themes were representative of common experiences described in the included studies. These provided a structure to systematically examine and discuss the evidence.

Included studies were from the United States (n=7) [20,37,39,41,44,50,52], United Kingdom (n=5) [38,43,47,48,51], Canada (n=2) [42,49], New Zealand (n=1) [40], France (n=1) [46], and Australia (n=1) [45]. Study methodology included in-depth or semistructured face-to-face interviews [38,40,42,43,48,49], surveys and questionnaires [20,44-48,50-52], focus groups [37,49], and analysis of online blog posts and comments [39,41]. Experiences with technologies examined included studies on CGM [38,39,44,49-52], FGM [46], CPGM [20], insulin pump therapy and bolus advisers [43], CSII [45], SAPT [42], and HCL/CL [37,48]. Some studies included experiences of using insulin pumps and/or CGM [40,41,47]. Study sample sizes ranged from 6 to 347, with participants comprised of parents and young people, with ages ranging from 4 to 24 years.

### Quality Assessment

The consensus rating for all studies on bias was low risk, and thus, none of the 17 studies needed to be excluded because of high risk of bias (Multimedia Appendix 2).

### Thematic Results

People’s experiences with devices were described within eight themes that included expectations prior to device use on one hand and usage experiences on the other hand. The themes were as follows: (1) expectations of the technology prior to use, (2) impact on sleep and overnight experiences, (3) experiences with alarms, (4) impact on relationships and independence, (5) perceived impact on blood glucose control, (6) device design and features (quality: equipment and size; data and trends: visualization, accuracy, and calibration; invasiveness), (7) cost, and (8) user satisfaction (Multimedia Appendix 3).

#### Expectations of the Technology Prior to Use

Adolescents expected HCL technology to be self-sufficient, believing it would provide a hands-off experience and live up to its name of an “artificial pancreas,” thereby giving them a break from managing diabetes [37]. Both parents and young people expected that HCL [37], SAPT/CGM/pump [41], and CPGM [20] would reduce the burden of diabetes in their lives. Prior to the use of CL technology, more than half of adolescents and parents reported an expectation of feeling safe when using CL systems, and some parents anticipated that their sleep would be better [48]. However, half of both groups anticipated a negative impact on their usual care routines [48]. At the same time, adolescents worried that CL would draw more attention to their diabetes [48].

Potential users of SAPT expected increased spontaneity and independence, feelings of normality, improved physical performance, and minimized SMBG, as well as reduced hypoglycemic and hyperglycemic episodes in adolescents [42]. Parents expected SAPT to simplify diabetes management and to enable a “normal” life for their child, while adolescents expected that CGM and insulin pump data sharing would reduce parental anxiety at night [40].

Parents believed that SAPT could serve as a second pair of eyes (safety mechanism), especially at night, and that it would help optimize the child’s glycemic control (as measured by HbA_1c_) to prevent future complications, alleviate stress in the parent-child relationship, and reduce their own anxiety [42]. In general, it was expected that CGM would make life easier for both parents and T1DM children [49], and excitement was expressed about new CGM and pump devices owing to expectations that they might reduce the T1DM management burden [41].

#### Perceived Impact on Sleep and Overnight Experiences

Seven studies reported results related to overnight device use, including studies on CGM [41,47,49-51], and CL [48] or HCL devices [37,48]. Young participants with T1DM using HCL/CL devices and their parents described waking up feeling better [48], with glucose levels in range [37,48], the benefits of which had an enduring positive effect throughout the day [48]. More stable blood glucose resulted in fewer alarms at night when using CL [48] or HCL [37], and reduced fear of hypoglycemia. Similarly, for (standalone) CGM systems, improved night-time diabetes management, a feeling of safety and reduced fear, and improved sleep were reported [38,49-51]. Easy access to sensor glucose levels at night increased knowledge [38] and resulted in improved self-management confidence [50].

Some parents in the Health Quality Ontario study [49] reported that despite known long-term risks, before using CGM, they had deliberately kept their child’s blood glucose level high before sleep to avoid overnight hypoglycemic episodes. The use of CGM had enabled better management decisions, including the cessation of this practice. Some parents in this and other studies about CGM stated that the device had saved their child’s life overnight [38,49,51]. Parents also reported disrupted sleep related to CGM due to either false alarms or fear of hypoglycemic events [41,47].

#### Alarms

Experiences reported about alarms referred to CGM [38,41,44,47,49,51,52], SAPT [42], and HCL systems [37]. Parents and young people reported a sense of reassurance and safety with CGM alarms, in the knowledge that they provided protection against hypoglycemic episodes [38,49]. Caregivers of children under 18 years of age using CGM found alarms useful in understanding the trending direction of glucose levels [51]. Both CGM [49] and HCL [37] device alarms were considered particularly useful for overnight management. A small number of young people and parents using CGM reported that alarms were the best thing about the device [52]. Users of an HCL system [37] reported fewer overnight interruptions from alarms due to fewer out of range glucose levels.

The benefits of alarms were accompanied by a variety of challenges. HCL users found responding to alarms burdensome [37]. In the Health Quality Ontario study, alarm fatigue amongst adolescents was reported as the most common barrier to the use of CGM [49]. Parents in two studies reported that their children found CGM alarms disruptive during school, which caused some young people to turn them off, impeding optimum diabetes management [38,51]. In one study, parents reported that their children felt nagged by CGM alarms and that they constituted a constant reminder of diabetes in their lives [38]. Interference in daily routine from CGM alarms was reported by more than one-third of participants in a study of young people aged 3 to 25 years [44]. For some parents, alarms were perceived as a sign of their own failure to achieve optimal glycemic control for their child [38].

Both parents and young people reported disrupted sleep related to CGM alarms. In a study of 100 parents of children with T1DM using CGM and insulin pumps [47], the majority of parents reported waking due to the technology, with more than half woken at least four times a week [47], and for one-third of these, the main reason was CGM alarms. Despite CGM alarms, one-fifth of these parents were still fearful of overnight hypoglycemia, and while false alarms were uncommon, they were reported by one-quarter of the parents [47]. Waking due to alarms was reported as frustrating for SAPT users because it was frequently unclear why they went off (whether it was serious or not) [42]. Moreover, alarms went off at inconvenient times and drew attention to the young person, which was perceived as embarrassing [42].

#### Perceived Impact of Device Use on Relationships and Independence

Eight studies on CL [48], HCL [37], CPGM [20], CGM [38-40,51], and SAPT [42] discussed the impact that devices had on relationships, and nine studies on CPGM [20], HCL [37], CGM [39,40,49,51], SAPT [42], FGM [46], and pump/bolus advisors [43] examined devices and independence of young people in their disease management.

Data sharing oscillated between providing a sense of independence and being a cause of conflict and resentment [39]. On one hand, adolescents and parents felt that SAPT [42], CGM [39,40,49,51], insulin pumps/bolus advisors [43], or CPGM [20] increased the young individual’s independence and autonomy in managing diabetes as parents did not have to be as hands on as before. This also reduced stress for parents [20] and allowed youth to participate in various leisure activities such as sleepovers, camps, and sports [43,51]. Young people were grateful for the capacity that CGM [40,51] and HCL [37] systems enabled for increased independence and better quality of life, boosting their confidence to try new things and to be more active [40,49,51]. The devices offered freedom to live life in near normality [40,49,51]. Parents also felt that CGM allowed their children to have a sense of safety and of not being alone [39]. Similarly, HCL was reported to result in improved relationships [37] and CL was reported to result in opportunities to talk to people about diabetes (owing to device visibility) [48].

On the other hand, experiences with SAPT included feelings of being tracked and spied on (adolescents) and fear of losing control (parents) [42]. One study that analyzed blogposts from 16 parents of children with T1DM reported that data sharing complicated relationships with a noticeable shift in dependence when adolescents learned to manage their diabetes and parental concerns were perceived as intrusive [39]. In another study about living with SAPT, while some parents reported a desire for their children to use SAPT for “their own peace of mind” [42], they also recognized the negative emotional impact on their child of being accountable for self-management 24 hours a day, and acquiesced to their child’s request to abandon the use of CGM as part of SAPT [42]. These reasons resulted in some parents and children deliberately refraining from sharing data or at least discussing the boundaries of data sharing [39,42]. Some teenagers preferred to share CGM data with friends they trusted rather than with their parents [39]. In general, parents referred more to partnerships than did young people, approaching management with CGM and insulin pumps as a team, encouraging, and cheerleading, although they were also aware that adolescents often perceived this as nagging [47].

#### Perceived Impact on Blood Glucose Levels

Participants in nine of the included studies reported that using technologies had a positive impact on blood glucose management [20,37,38,44,46-49,51]. Steadier blood glucose levels were reported when using HCL [37], and improved blood glucose control was noted with CL [48] and CGM use [44,49,51], with reduced frequency and severity of hypoglycemic events in CGM users [47], as well as lower HbA_1c_ levels when using CPGM [20] and FGM [46]. The majority of caregivers surveyed about the use of both CGM and CSII reported improvements in achieving glycemic targets [47]. Users reported greater confidence and reassurance (CL) [48], and better management decisions (CGM) [49]. Better management also meant less likely over-correction of lows/highs (CGM) [38]. Reduced hypoglycemia-related anxiety was one of the most common perceived benefits of CGM [44]. Overall, parents described CGM as an empowering and motivating tool to fine-tune blood glucose control [38].

#### Experiences Related to Device Design and Features

Participants in 15 studies discussed device design features in terms of device quality [20,38,40-46,48,49,51,52], data characteristics [20,37-42,44,46,48,49,51,52], and discomfort [40,42,44,46,49,51,52].

##### Device Quality: Equipment and Size

One commonly reported disadvantage of CGM [40,44,49,52], SAPT [42], and CL [48] was bulky and heavy sensors and devices. Adolescents experienced challenges with device size and visibility to peers, and described SAPT devices as “ugly” [42]. Managing and wearing additional devices, with increased responsibility, workload, and “hassle,” were reported as parental concerns for CGM [49,51] and SAPT [42], and for young people, it was a constant reminder of living with T1DM [40,49]. In addition, participants did not like the need for CGM backup equipment [40] or second cannulas for CL systems [48].

CGM sensor failures and technical problems, such as sensor cut out and false low values when sleeping on the sensor, were reported [51], in addition to poor FGM [46], HCL [37], and SAPT [42] sensor adhesion (additional tape needed to secure devices) [46] and CGM buttons or power port covers falling off [41]. Children and adolescents had mostly positive experiences with CSII and planned to continue its use as adults [45]. Young people liked that pumps did not require multiple insulin injections [40].

##### Data Trends

Data trends and graphs allowed visualization of changing glucose levels, which made CGM superior to SMBG [38], made understanding CPGM trends easier for youth [20], allowed parents to adjust dosage immediately [49], enabled CGM users “to self-correct out-of-range glucose levels” [52], and translated retrospective CGM data analysis into better understanding of diabetes for informed future decisions [38,51]. Yet, constant streaming of CGM data was described as overwhelming at times, and parents and children found that they needed to establish a routine for using the data [39,49,51]. Difficulties interpreting CGM [51] and SAPT [42] data and graphs were also reported. One study of young people’s use of CL reported that parents found greater value in the graphs and trends than did adolescents (CL) [48].

##### Data Lag

Device accuracy and the paradox of inaccurate data due to lag time between the interstitial and capillary blood glucose levels was a key challenge for one-quarter of FGM users [46], with some choosing to discontinue use because of this [46]. The data lag time created a feeling of data distrust for users of CGM [38,51] and SAPT [42], who resorted to SMBG to clarify high and low readings [38,42,51]. Data distrust caused frustration for adolescents who had previously relied on their embodied experiences to understand blood glucose levels but began doubting their decision-making ability [40,42]. Other studies reported that caregivers thought CGM had good data accuracy [41] or that CPGM data were accurate [20].

##### Connectivity and Calibration

Parents of young users of CL reported that connectivity and device calibration were the worst aspects of use [48]. Recalibration was perceived as a burden or as frustrating by CGM [38,52], SAPT [42], CL [48], and HCL [37] users. In addition to calibration, users of HCL technology found that the amount of information to be entered about meals, boluses, and corrective insulin dosages was burdensome [37].

##### Discomfort Related to Devices

Young people reported that the insertion of CGM [38,44,51,52], SAPT [42], and FGM [46] sensors was painful or irritating. For some CGM/pump [38,49] and FGM [46] users, this resulted in reluctance for both future insertion and removal of the sensor, and in discontinued device use [46]. Yet, reduced finger pricking was seen as an advantage of CGM [40,51] and sometimes was the motivation to use new technology (eg, FGM) [46]. Overall, complaints about CGM (including calibration, size, and difficulty inserting the device) were tempered with an emphasis on the benefits users experienced, which they believed outweighed any disadvantages [38,51].

#### Financial Cost

Four studies from New Zealand [40], Canada [42,49], and the United Kingdom [51] considered the financial cost of SAPT/insulin pumps and CGM devices. Cost issues were cited as the main reason for interrupting or ceasing FGM use in a French study [46] and as a reason for not using CPGM in the United States [20]. Parents and adolescents were described as “living worried,” being faced with the stressor of reconciling affordability of SAPT devices with everyday living costs [42]. Parents reported that CGM/SAPT was too expensive to fund themselves owing to the high ongoing supply requirements [42] and the short life span of replaceable sensors [49]. Some used CGM sensors longer than recommended to save money [49]. In Canada, lack of insurance and/or government funding for CGM compared to insulin pumps was cited as a barrier to uptake [42,49]. If asked to choose between an insulin pump and CGM, some parents opted for CGM since they considered continuous data and information more valuable than the flexibility offered by a pump [49].

#### Satisfaction With the Technology

One US study of 208 youth aged 8 to 18 years and their parents [52] measured satisfaction using the Continuous Glucose Monitoring Satisfaction Scale (CGM-SAT), which includes 5-point Likert subscales on the “benefits of CGM” and “hassles of CGM.” Parents’ and adolescents’ responses were compared, as was CGM use in terms of days per week. Overall, satisfaction with CGM technology was higher for parents compared to young people [52]. Frequent users who used CGM for over 6 days per week reported considerably higher satisfaction compared with those who used CGM for less than 4 days per week [52]. In another US study, among 35 families using the mySentry CGM system [50], parents reported high levels of satisfaction with overnight monitoring of their child’s glucose levels. In a French study of 347 FGM users aged 0 to 18 years, overall satisfaction was high, with two-thirds of users reporting being satisfied [46]. The most frequent motive for dissatisfaction with FGM was the absence of real-time alerts [46]. Regarding CL technology, overall, there were favorable responses in terms of impact and satisfaction [48].

## Discussion

### Principal Findings

The eight themes that emerged from our review of the 17 included studies illustrate the impacts of diabetes and the associated use of technology on various aspects of young people’s and their caregivers’ lives.

Our results showed that expectations prior to technology use could be split into expectations that could not be met with the current state of the technology (eg, artificial pancreas [37]) and expectations that were pretty much mirrored by the reported experiences (eg, improved safety). Experiences partly depended on the particular technology used. The majority of the papers focused on CGM and/or insulin pumps, with some reporting experiences specific to the respective devices (eg, CGM sensor accuracy/failure). However, as the results for CGM and insulin pumps are frequently reported together, further research is needed to examine if the difference in the devices is a key factor in user experiences.

Sleep disturbances due to alarms in youth and caregivers, and overnight management have been reported as major challenges in T1DM management in previous research [53], along with anxiety and fear of hypoglycemia in both youth and their caregivers [54]. Efficient and reliable hypoglycemia alert systems that do not disrupt sleep to an extent that affects overall management still have to be developed.

While parents are solely responsible for disease management of young children, the dynamics of care coordination change in adolescence, requiring fine balancing of parental support and involvement [11]. Adolescence is a time when children seek to achieve increasing independence and to separate emotionally from their parents, prioritizing relationships with their age peers. During this time, diabetes can impact the many important relationships of young people, including relationships with their parents, health professionals, teachers, and peers [20]. Our results indicate that automatized monitoring systems and insulin pumps offer potential for greater independence in adolescents and reduce the ongoing monitoring and management burden for parents [55]. At the same time, technologies can negatively affect the relationship between adolescents and their caregivers (eg, data sharing complicates relationships). Young people’s expectations of technology often diverge from those of their caregivers, and priorities are set differently (eg, independence versus reduced fear of hypoglycemia and improved sleep). Moreover, stigmatization [56] and judgement [57] by family members or peers can affect relationships and overall diabetes management. Thus, the nature of relationships between young people with T1DM and their caregivers, peers, and health professionals needs to be accounted for in the design of these technologies, particularly the relationship between youth with T1DM and their parents, which is characterized by a fine balance between autonomy and dependence (interdependence, also termed as transactional) [58]. Reliable devices are needed to engender trust and encourage practices that optimize diabetes management, avoiding risky behaviors that were reported by some participants in this review (eg, parents allowing higher than desirable blood glucose levels to avoid overnight hypoglycemia) [59].

Diabetes technology has been shown to be effective in improving metabolic control [6] in young people with T1DM at an early stage of the disease, preventing long-term complications (referred to as “metabolic memory”) [60]. Similar to studies of CGM, HCL, and CL in our review, previous research has found that technology can improve the quality of life of children and adolescents [6]. Technology holds potential to facilitate self-management in a way that reduces the effects of the disease on daily life, balancing daily activities with diabetes self-management demands and decreasing psychological pressure, stressors, and fear [61]. This holds great promise for adolescents, a high proportion of whom are distressed about diabetes and thus have suboptimal diabetes outcomes [62,63].

Successful diabetes technology use and improved self-care, which are reflected in improved blood glucose levels, can be achieved when individual empowerment is promoted [64,65]. Thus, a particular focus should be put on empowerment practices when designing diabetes technology for self-management. This can be achieved through user-centric design, which can aid in removing barriers to use at the same time, enabling the development of systems that are suitable for long-term use [66]. User expectations and preferences in technology design need to be accounted for (eg, reduction in device size and improved device quality as mentioned in our review).

Cost and funding issues hindered technology uptake and potential T1DM self-care in the included studies. While government subsidies are available for blood glucose meters in New Zealand, users in our review reported frequent changes by the government, which forced them to acquire newer and cheaper devices more prone to inaccurate measurements. Lack of insurance and/or government funding for CGM systems in Canada and the United Kingdom, and for CPGM systems in the United States [20] has been reported as an uptake barrier in the studies included in our review. FGM became reimbursable in France under the French National Health Insurance program in 2017 [46]. In Australia, subsidized schemes of CGM for children and adolescents have been expanded by the government to include FGM starting from 2020, but for many, these schemes cut out at the age of 21 years [67]. This shows that funding for new diabetes technology varies widely among countries, impacting technology uptake and use.

Despite a variety of reported challenges in using technologies to manage T1DM, overall, the studies in our review examining satisfaction with use reported high levels of satisfaction, and benefits were predominant. This is congruent with previous research that found new technology use is frequently accompanied with increased satisfaction with the technology when compared to multiple dose injections and SMBG [68].

Owing to its perceived benefits, there is a growing desire among the young T1DM community for automated CL “artificial pancreas systems” that integrate CGM with insulin delivery [69]. Yet, these expectations and desires are frequently not met in actual experiences with available technology. Even though available systems are a step toward automation of diabetes control, our review demonstrates that current technology is insufficient to provide fully reliable and sustainable automated systems that fulfill the expectations of young people with diabetes and their caregivers. The gap between “ideal” device systems, such as CL systems (artificial pancreas), and the currently available status quo of systems (eg, sensors and HCL systems) is a barrier that warrants further development. There is a need for improved and advanced diabetes technologies complying with the various user requirements outlined above.

The strength of this review lies in its unique focus on young individuals with T1DM, as this population is among those that experience what has been identified as “diabetic distress” and that undergo the most difficulty in adapting to diabetes needs and are most challenged in terms of glycemic variability [63].

### Implications for Practice

The conglomeration of experiences and attitudes associated with currently available diabetes devices and technologies is a step toward a possible refinement of future diabetes technologies. Our review supports a move toward a tailored approach for individuals with T1DM to create technology that is robust, intuitive, and sustainable. An integrative approach involving adolescents, parents, health care providers, and teachers should be used to develop future technology and guide design experiments. Individuals with T1DM from diverse ethnic and socioeconomic backgrounds also need to be included in the co-design process to advance T1DM technology. This includes discussions of use and sharing of data. Our review has shown that while access to continuous data was valued by CGM users, there were also challenges in managing the amount of data. This resonates with a clinical evidence review of 22 studies that found that data could be perceived as overwhelming for some users [49]. Challenges like these must be addressed in collaboration with young people with T1DM and their caregivers.

### Study Limitations

While our main interest was in examining adolescents’ and their caregivers’ experiences of using devices, some included studies also involved younger children and older youth. It was not possible to exclude these data from our analysis, and at times, these have been included in our analysis.

We did not examine the grey literature, and thus, we might have excluded reports and evaluations that also included experiential data. We only examined studies reported in English, which excludes analysis of experiences in non–English-speaking countries and perhaps young non–English-speaking people’s experiences of using devices in English-speaking countries.

Owing to the rapid evolution of technology and associated changes regarding available devices and systems, there are challenges in evaluating a large number of experiences with a particular device.

### Conclusion

Overall, the use of diabetes technology was found to be beneficial and to positively impact disease management for both young people and their caregivers. The included studies reported the advantages of diabetes technologies, such as improved self-management and diabetes outcomes, in young people associated with improved monitoring, data tracking, and data sharing, as well as decreased anxiety and psychological pressure in both parents and children. However, technology did not always live up to users’ expectations. Several barriers and challenges toward its use were reported, such as cost, the size and visibility of devices, and the intrusiveness of alarms, which drew attention to the fact that the user had T1DM. Continued use of diabetes technology was underpinned by its benefits outweighing its challenges, especially among younger people. Collaboration with young people and their caregivers is essential to ensure that future T1DM technologies meet their expectations and needs.

## Appendix

Multimedia Appendix 1 Data extraction table of included studies.

Multimedia Appendix 2 Quality assessment using the Mixed Methods Appraisal Tool (MMAT).

Multimedia Appendix 3 Themes derived from included studies.

## Abbreviations

CGM: continuous glucose monitoring
CL: closed loop
CPGM: cell phone glucose monitoring
CSII: continuous subcutaneous insulin infusion
FGM: flash glucose monitoring
HCL: hybrid closed loop
SAPT: sensor-augmented pump therapy
SMBG: self-monitoring of blood glucose
T1DM: type 1 diabetes mellitus
