# Supplementary material for: Experiences of Young People and Their Caregivers of Using Technology to Manage Type 1 Diabetes Mellitus: Systematic Literature Review and Narrative Synthesis
Source: JMIR Diabetes. 2021 Feb 2;6(1):e20973. doi: 10.2196/20973 (PMC7886614; doi:10.2196/20973)
Supplement: Multimedia Appendix 1 [file diabetes_v6i1e20973_app1.docx]

**Multimedia Appendix 1.** Data extraction table of included studies

| Author Year  Country | Population | Technology | N | Age groups | Aims | Study design | Key Findings | Themes |
| --- | --- | --- | --- | --- | --- | --- | --- | --- |
|  |  |  |  |  |  |  |  |  |
| Barnard et al (2016) [47]  UK | Partners and caregivers of people with T1DM | CGM &/or insulin pump | 174 | Adults >18 years | To explore the impact of T1DM-related technology | Online questionnaire survey including open and closed questions (e.g., PAID-5, WHO-5) | 86% partners, 82% parents/caregivers reported technology had made it easier to achieve blood glucose targets. Diabetes-related distress was common, as was sleep disturbance associated with device alarms/fear of hypoglycemia. Reduced frequency and severity of hypoglycemia related to device use reported by 50% of participants. | Night, alarms, blood glucose levels, satisfaction |
| Barnard et al (2017) [48]  UK | Adults and children/  Adolescents with T1DM using insulin pump therapy | CL | 58 | Adults ≥18 years, children/ adolescents 6-18 years | To explore psychosocial experiences of closed loop technology for adults, children, and  adolescents with T1DM and their parents | Open-label, randomized, two-period crossover design: 12 weeks of automated CL glucose control, then 12 weeks of SAPT/open loop, or vice versa; semi-structured interviews | Moderately favorable impact of, and satisfaction with, both open and closed loop interventions, but little evidence of a comparative advantage of either. | Expectations, night, relationships,  blood glucose levels, device quality, data, trends, satisfaction, other |
| Carroll et al (2011) [20]  USA | Adolescents with T1DM | Cell-Phone Glucose Moni-toring (Glucophone) | 39 | 13-18 years | To assess the feasibility and acceptability of a cell phone glucose monitoring system for adolescents with T1DM and their parents | System use for 6 months, filling out surveys every 3 months | Adolescents reported positive feelings about the technology, despite significant technical issues that affected continued use. Nearly all thought that the clinic involvement in monitoring testing behavior was acceptable. The use of the Glucophone™ did not significantly change the quality of life, level of conflict with parents, reported self-management, or average glycemic control. | Relationships, independence, blood glucose levels, device quality, data, cost, other |
| Cemeroglu et al  (2010)  [44]  USA | T1DM patients/  caregivers with interest in RT-CGMS and on insulin pump therapy | CGM (& insulin pump) | 43 | 3-25 years | To assess patient and caregiver’s perception of benefits and disadvantages of RT-CGMS in children or young adults with T1DM on insulin pump therapy | 4-week trial of RTGMS with short-term usage group versus long-term usage group (2 months or longer on RT-CGM) followed by a survey questionnaire | Hypoglycemia prevention was the most common perceived benefit (88%), followed by elimination of hypoglycemia-related anxiety (83%), ease of pattern management (85%), improvement of diabetes control (80%), improvement of quality of life (78%), and ease of diabetes care (78%). Negative effects included irritation/annoyance from the sensor alarm (48%) and insertion site bruising, pain, or irritation (43%). | Alarms, blood glucose levels, device quality, trends, invasiveness, other |
| Iturralde et al (2017) [37]  USA | Adults and adolescents with T1DM and 4-5 days of HCL use | HCL | 32 | Adults 28.2 ± 6.1 years, adolescents 16.6 ± 0.9 years | To explore the benefits, expectations, and attitudes of individuals with T1DM following a clinical trial of an HCL system | Focus groups  after 4 to 5 days of HCL system use | Some participants felt misled by terms such as “closed loop” and “artificial pancreas,” which seemed to imply a more “hands-off” experience. Perceived benefits were improved glycemic control, anticipated reduction of long-term complications, better quality of life, and reduced mental burden of diabetes. Hassles and limitations included unexpected tasks for the user, difficulties wearing the system, concerns about controlling highs, and being reminded of diabetes. | Expectations, night, alarms, relationships, independence, blood glucose levels, data, other |
| Kaiserman et al  (2013)  [50]  USA | Families with a child with T1DM | CGM: my-  Sentry (& insulin pump) | 35 | 11.9 ± 2.70 years | To evaluate the  mySentry system’s usability, acceptability, and impact on nocturnal glycemic consequences in families with a child or adolescent with T1DM | Baseline survey, 1-week run-in phase, use of mySentry system for 3-weeks, post-survey, narrative comments, daily night logs | Most parents were fearful of their unawareness of their children’s nocturnal glucose excursions. The mySentry system met the predefined acceptability criteria for general experience, product usability, and training materials. There were no unanticipated device-related adverse effects. Among children who experienced nocturnal hypo- or hyperglycemic episodes there was a trend toward less frequent and less prolonged episodes during mySentry use. | Night, satisfaction, other |
| Lawton et al (2018) [38]  USA | Adults & adolescents with T1DM who had used CGM, parents of children with T1DM & CGM use | CGM (& insulin pump) | 24 | ≥16 years, 13–  15 years (plus parents/caregivers), parents/caregivers of individuals ≤12 years | To explore participants’ experiences of using CGM | In-depth interviews (inductive approach guided by grounded theory) | Participants found CGM an empowering tool, aiding lifestyle planning and enabling to take action. Easy continuous data access allowed to develop a better understanding of impacts on blood glucose. CGM data provided a more nuanced picture of blood glucose control. Participants expressed confidence, but most described expecting health professionals to interpret CGM data. While alarms could reinforce a sense of safety, some individuals expressed ambivalent views. | Night, alarms, relationships, blood glucose levels, device quality, data, trends |
| Litchman et al (2018) [39]  USA | Adults with T1DM, parents of T1DM children, spouses | CGM | 39 blogs (206 com-ments) | Adults, parents of T1DM children of all age groups | To identify the benefits and challenges related to RT-CGM data sharing from the patient and care partner perspective and to explore the number and type of individuals who share and follow RT-CGM data | Content analysis of 39 publicly available blogs with 206 comments focused on RT-CGM and data sharing | The analysis resulted in three themes: (1) RT-CGM data sharing enhances feelings of safety, (2) the need to communicate boundaries to avoid judgment, and (3) choice about sharing and following RT-CGM data. | Relationships, independence, data |
| McCarthy et al (2017) [40]  New Zealand | Young females with T1DM | Various includ-ing CGM, insulin pump | 6 | 13-24 years | To trial a letter writing method for eliciting female adolescents’ wide-ranging user requirements for the self-management of T1DM | Case study: Participants wrote letters to one of their medical devices,  follow-up interviews | Four categories: acquiring and changing medical devices, requiring convenience and practicality for everyday contexts, collecting and using data, and corresponding with preferences and values. Young people are often excluded from research and development regarding medical devices, yet study method was successful in identifying experiences and preferences to inform the design of medical devices. | Expectations, relationships, independence, device quality, data, invasiveness, cost |
| Ontario Health Quality (2018)  [49]  Canada | People with T1DM or parents of T1DM children | CGM | 59 | Adults, children 2-16 years | To explore the underlying values, needs, impacts, and preferences of those who have lived experience with T1DM, with a focus on CGM versus usual care | Face-to-face interviews, phone interviews, written interview responses, focus groups; (+ clinical evidence, economic impact studies, not focused on) | Adult patients and parents of children with T1DM reported very positive experiences with CGM. The high ongoing cost of continuous glucose monitoring devices was seen as the greatest barrier to their widespread use. | Expectations, night, alarms, independence, blood glucose levels, device quality, data, trends, invasiveness, cost, satisfaction |
| Oser et al (2017)  [41]  USA | Care-givers to T1DM children | CGM &/ or insulin pump | 3 blogs (140 posts & 663 comments) | 4-16 years | To analyze blogs of caregivers to children with T1DM and to assess the blogs for the presence of unsafe or inaccurate clinical information | Content analysis of blog posts and comments | Five major themes: 1) impact of the diagnosis, 2) burden of intense self-management, 3) caregivers’ use of technology to ease their fear of hypoglycaemia and impacts that alarms have on caregiver burden, 4) caregivers’ perceptions of missed or delayed diagnosis (frustration), and 5) resilience that caregivers develop despite the burdens. Caregivers find support through advocacy efforts and peer-to-peer blogging. | Expectations, night, alarms, device quality, data |
| Perry et al (2017)  [45]  Australia | Young people with T1DM and their parents/  guardians | CSII | 107 | 12-18 years | To explore young people's attitudes, perceptions, and experiences with diabetes management comparing those using/not using CSII, and proportions likely to transition to adult services requiring initiation and/or support for CSII use | Cross-sectional survey  (e.g., Perceived Diabetes Self-Management  Scale, PDSMS) | Positive attitudes and perceptions of self-efficacy and diabetes management, but moderate disturbance by diabetes and experiences of suboptimal management outcomes. Patterns of associations were demonstrated between knowledge, attitudes, and experiences of diabetes modelled by regression analysis. There were no statistically significant differences in responses between users and nonusers of CSII. Over 40% indicated their intention to use the technology as adults. | Blood glucose levels, device quality, satisfaction, other |
| Pickup et al  (2015)  [51]  USA | Adults with T1DM and caregivers of children with T1DM using CGM | CGM | 100 | Adults >18 years of children <18 years | To understand the range of user perceptions and experiences of real-time CGM use in T1DM | Online survey, including free-text patient narratives | Four themes: 1) metabolic control, 2) living with CGM, 3) psychological issues and patient/caregiver attitudes, and 4) barriers to CGM use. Despite hassles, experiences were positive, with improved glycaemic control, diet/exercise management, quality of life, and well-being. Technical problems included sensor inaccuracy and unreliability, and “alarm fatigue”. Advantages of CGM used with an insulin pump with automatic insulin suspension during hypoglycaemia were recorded by several participants. | Night, alarms, relationships, independence, blood glucose levels, device quality, data, trends, invasiveness, cost, satisfaction, other |
| Rankin et al (2018) [43]  UK | Children with T1DM | Insulin pump &/ or bolus advisors | 24 | 9-12 years | To explore the challenges pre-adolescent children encounter when self-managing diabetes and the factors which motivate and enable them to take on new diabetes-related tasks | In-depth interviews using age-appropriate questioning | Children reported several barriers to self-management tasks. Several discussed being motivated to take on management responsibilities when they started secondary school. They felt enabled to take on responsibilities by using strategies which limited the need to perform complex maths. Many discussed using bolus advisors with pre-programmed ratios and entering values provided by their parents to calculate insulin doses. Several described using mobile phones to seek advice about carbohydrate contents in food. | Independence, blood glucose levels, device quality |
| Rashotte et al (2014) [42]  Canada | Adolescents with T1DM with SAPT experience, parents | SAPT | 16 | 13-17 years | To explore adolescents’ and parents’ daily experience of living with SAPT | Interpretive phenomenological study design: in-depth, digitally recorded interviews | The overarching theme, seeking harmony, reflected adolescents’ and parents’ struggles with balancing tensions that arose from managing SAPT, while also struggling to live with both wellness and chronic illness. Four themes constituted the struggle to find harmony: struggling with hopes and expectations for SAPT, being ready for SAPT, living the burdens of continuous glucose monitoring and creating partnerships. | Expectations, alarms, relationships, independence, device quality, data, invasiveness, cost, other |
| Tansey et al  (2011)  [52]  USA | T1DM people over 8 years old on insulin pump or multiple daily injections | CGM (& insulin pumps or MDIs) | 432 | Adults ≥ 18 years, children 8 to <18 years | To assess satisfaction with CGM in T1DM | Survey of users using Continuous Glucose Monitoring Satisfaction Scale after 6 months, open-ended questions | More frequent monitoring was associated with higher satisfaction for adults (n= 224), youths (n= 208) and parents of youths (n= 192) (all P< 0.001) in the sub-scales, but the greatest differences between the groups involved scores on hassle items. Common barriers to monitoring use included insertion pain, system alarms and body issues; while common benefits included glucose trend data, opportunities to self-correct out-of-range glucose levels and to detect hypoglycaemia. | Alarms, device quality, data, trends, invasiveness, satisfaction |
| Vergier et al (2019) [46]  France | Children with T1DM using FreeStyle  Libre ® FGM system and/or parents | FGM | 347 | 0-18 years | To understand the usage experiences of children with T1DM using FGM | Retrospective multicenter evaluation of patient and family opinions: survey | 79.5% had been using the sensor for more than three months. The main motivations were to avoid finger prick pain and to allow parents to check nocturnal glucose levels. Two-thirds of respondents experienced difficulties, mainly the sensor falling off, measurement discrepancies and cutaneous reactions; 89.5% changed their habits: 70.6% took more scans, 37.2% corrected their hyperglycaemia more promptly, and 37.5% used trends to adjust their insulin dosage. About one-third experienced lower HbA1c levels, and two thirds were satisfied with the device. | Expectations, independence, blood glucose levels, device quality, data, trends, invasiveness, cost, satisfaction |
