# Supplementary material for: Experiences of Young People and Their Caregivers of Using Technology to Manage Type 1 Diabetes Mellitus: Systematic Literature Review and Narrative Synthesis
Source: JMIR Diabetes. 2021 Feb 2;6(1):e20973. doi: 10.2196/20973 (PMC7886614; doi:10.2196/20973)
Supplement: Multimedia Appendix 2 [file diabetes_v6i1e20973_app2.docx]

**Multimedia Appendix 2.** Quality assessment using the Mixed Methods Appraisal Tool (MMAT)

| Study | Reviewer | Question 1 | Question 2 | Question 3 | Question 4 | Question 5 | Screening  question 1 | Screening  question 2 | Low Bias | Unclear Bias | High Bias |
| --- | --- | --- | --- | --- | --- | --- | --- | --- | --- | --- | --- |
| Qualitative | | | | | | | | | | | |
| Iturralde (2017) [37] | NBS | low | low | low | low | low | low | low |  |  |  |
|  | MC |  | low | low | low | low |  | low |  |  |  |
|  | Consensus | low | low | low | low | low | low | low | 7 | 0 | 0 |
| Lawton (2018) [38] | NBS | low | low | low | low | low | low | low |  |  |  |
|  | MC | low | low | low | low | low | low | low |  |  |  |
|  | Consensus | low | low | low | low | low | low | low | 7 | 0 | 0 |
| Litchman (2018) [39] | NBS | unclear | low | low | low | low | low | low |  |  |  |
|  | MC | low | high | low | low | low | low | low |  |  |  |
|  | Consensus | unclear | unclear | low | low | low | low | low | 5 | 2 | 0 |
| McCarthy (2017) [40] | NBS | low | unclear | low | low | low | low | unclear |  |  |  |
|  | MC | low | low | low | low | low | low | low |  |  |  |
|  | Consensus | low | unclear | low | low | low | low | low | 6 | 1 | 0 |
| Oser (2017) [41] | NBS | low | low | low | low | low | low | low |  |  |  |
|  | MC | low | low | low | low | low | low | low |  |  |  |
|  | Consensus | low | low | low | low | low | low | low | 7 | 0 | 0 |
| Rankin (2018) [43] | NBS | low | low | low | low | low | low | low |  |  |  |
|  | MC | low | low | low | low | low | low | low |  |  |  |
|  | Consensus | low | low | low | low | low | low | low | 7 | 0 | 0 |
| Rashotte (2014) [42] | NBS | low | low | low | low | low | low | low |  |  |  |
|  | MC | low | low | low | low | low | low | low |  |  |  |
|  | Consensus | low | low | low | low | low | low | low | 7 | 0 | 0 |
| Quantitative descriptive | | | | | | | | | | | |
| Carroll (2011) [20] | NBS | low | unclear | low | low | unclear | unclear | low |  |  |  |
|  | MC | low | low | low | low | low | low | low |  |  |  |
|  | Consensus | low | unclear | low | low | unclear | unclear | low | 4 | 3 | 0 |
| Cemeroglu (2010) [44] | NBS | low | unclear | low | unclear | unclear | low | low |  |  |  |
|  | MC | low | low | low | low | low | low | low |  |  |  |
|  | Consensus | low | unclear | unclear | unclear | unclear | low | low | 3 | 4 | 0 |
| Perry (2017) [45] | NBS | low | low | low | unclear | low | low | low |  |  |  |
|  | MC | low | low | low | unclear | low | low | low |  |  |  |
|  | Consensus | low | low | low | unclear | low | low | low | 6 | 1 | 0 |
| Vergier (2019) [46] | NBS | low | low | unclear | unclear | low | low | low |  |  |  |
|  | MC | low | low | low | unclear | low | low | low |  |  |  |
|  | Consensus | low | low | unclear | unclear | low | low | low | 5 | 2 | 0 |
| Mixed methods | | | | | | | | | | | |
| Barnard (2016) [47] | NBS | unclear | low | low | low | low | low | low |  |  |  |
|  | MC | low | low | unclear | high | low | low | low |  |  |  |
|  | Consensus | low | low | unclear | unclear | low | low | low | 5 | 2 | 0 |
| Barnard (2017) [48] | NBS | low | low | low | unclear | low | low | low |  |  |  |
|  | MC | low | low | low | low | low | low | low |  |  |  |
|  | Consensus | low | low | low | low | low | low | low | 7 | 0 | 0 |
| HealthQuality Ontario (2018)^a^ [49] | NBS | low | low | low | low | low | low | low |  |  |  |
|  | MC | low | low | low | low | low | low | low |  |  |  |
|  | Consensus | low | low | low | low | low | low | low | 7 | 0 | 0 |
| Kaiserman (2013)^b^ [50] | NBS | low | unclear | low | low | low | low | low |  |  |  |
|  | MC | low | low | low | low | unclear | low | low |  |  |  |
|  | Consensus | low | unclear | low | low | low | low | low | 6 | 1 | 0 |
| Pickup (2015) [51]^c^ | NBS | low | low | low | low | low | low | low |  |  |  |
|  | MC | unclear | low | low | unclear | low | low | low |  |  |  |
|  | Consensus | low | low | low | low | low | low | low | 7 | 0 | 0 |
| Tansey (2011) [52] | NBS | low | low | unclear | low | unclear | low | low |  |  |  |
|  | MC | low | low | low | unclear | low | low | low |  |  |  |
|  | Consensus | low | low | unclear | low | unclear | low | low | 5 | 2 | 0 |

*Notes*.

^a^ only qualitative patient study assessed (clinical evidence and economic impact studies not included in the review)

^b^ only quantitative data reported and assessed accordingly

^c^ only qualitative data reported and assessed accordingly
