# Supplementary material for: Experiences of Young People and Their Caregivers of Using Technology to Manage Type 1 Diabetes Mellitus: Systematic Literature Review and Narrative Synthesis
Source: JMIR Diabetes. 2021 Feb 2;6(1):e20973. doi: 10.2196/20973 (PMC7886614; doi:10.2196/20973)
Supplement: Multimedia Appendix 3 [file diabetes_v6i1e20973_app3.docx]

**Multimedia Appendix 3.** Themes derived from included studies

| **1.Expectations prior to use** | |
| --- | --- |
| Young people | - Self-sufficient technology – hands off (HCL [37]) - Reduce burden of managing diabetes (HCL [37]) - Freedom, spontaneity and independence (SAPT [42]) - Hope to minimise hypo- and hyper-glycaemic episodes (SAPT [42]) - To relieve overnight concern for parents, who could easily check BGLs (CGM [40]) |
| Parents | - Improve self-management (CGM [39], CPGM [20]) - Ease parental burden (CGM [39, 41], insulin pump [43]) - Second pair of eyes; reduce errors and prevent complications (SAPT [42]) - Overnight safety net (SAPT [42], CGM [49]) - Decrease anxiety (CGM [49], SAPT [42]) - Improve parental-child relationship (SAPT [42], CGM [49], CPGM [20]) |
| Both | - Reduce burden of managing diabetes (HCL [37], SAPT [42], CPGM [20]) - Reduce relational conflict (CPGM [20]) |
| **2. Perceived impact on sleep and overnight experiences** | |
| Young people | - Faster glucose measurements (FGM [46]) - Less frequent and less prolonged episodes of nocturnal hypo- or hyperglycaemia (CGM [50]) - Children described feeling better rested on waking (CGM [49]) - The benefits of waking with good morning glucose levels had an enduring positive effect throughout the day (CL [48]) |
| Parents | - Increased awareness of and confidence in managing overnight BGLs (CGM [50], CL [48]) - Faster, more convenient overnight checks by parents (CGM [40, 51], FGM [46]) - Improved sleep (CL [48], HCL [37], CGM [50, 51]) - Improved feelings of safety and reduced fear of hypoglycaemia (CL [48], CGM [51]) |
| Both | - Improved detection of overnight hypoglycaemia (CGM [49, 51], HCL [37], CL [48]), including saving lives (CGM [49, 51]), improved sleep for everyone (CGM [49-51]) |
| **3. Experiences with alarms** | |
| Young people | - Alarm fatigue amongst adolescents was the most common barrier to use of CGM [49] - Disruptive during school (CGM [38, 51]) - CGM [51] and SAPT [42] alarms intruded on daily activities - Distinguishing between SAPT alarms that could be ignored and those that were urgent [42] |
| Parents | - Fatigue related to alarms was common (CGM and insulin pumps [47]) - A sign of personal failure to achieve optimal glycaemic control (CGM [38]) |
| Both | - Psychological reassurance and sense of safety (CGM [38, 49]) - Assistance with overnight management (HCL [37], CGM [49]) - Fewer interruptions by alarms overnight (HCL[37]) - Disrupted sleep for both parents and children (CGM [41, 47, 49, 51, 52]) - While 10% of parents reported false alarms more than once per week (CGM [47]), they also reported positive impact of technology for their child |
| **4. Impact on independence and relationships** | |
| Young people | - Assisted young people who were previously reliant on parents for mathematical calculations, understanding carbohydrates and insulin boluses (insulin pump and bolus adviser [43], SAPT [42]) - Adolescents’ relationships with friends, teachers, coaches and health care providers required recognition of the seriousness of diabetes and responsible and open acceptance of SAPT [42] - Empathy and a sense of partnership with close friends enhanced integration of SAPT into daily life [42] - Increased independence and ability to pursue sports (CGM [51], HCL [37]) - Boosting confidence to try new things, and to be more active (CGM [40]) - Adolescent autonomy, starting high school (insulin pump [43]) - Alleviating parental burden (FGM [46], SAPT [42]) - Improved quality of life (CGM [49], HCL [37], (CL) [48]) |
| Parents | - The capacity to monitor data remotely was viewed positively by most parents, who felt it enabled them to give their children more freedom (CGM [49]) - Parental peace of mind (SAPT [42]), alleviating anxiety, making them more comfortable and confident with its use (CGM [40, 49, 51] and CL [48]) - Parents reported approaching management as a team, encouraging, cheerleading – all the things that teenagers consider as nagging (CGM and insulin pumps [47]) - Parents motivated by children’s wellbeing and despite their perceptions of benefit, accepted children’s preference not to use SAPT due to the burden of managing technology 24/7 [42] |
| Health care providers | - Healthcare professionals supportive with decisions about the technology, although the manufacturer’s hotline staff were crucial for daily technical support (SAPT [42]) - Important for interpreting historical CGM data and deciding on changes to insulin doses and food intake (CGM [38]) - Some healthcare professionals were perceived as unsupportive of adolescents’ use of CGMs, which was thought to be due to a lack of knowledge and expertise (CGM [49, 51]) - In some instances, parents reported turning to social media and peer-support to make up for information they required before they began using a CGM [49] |
| Schools | - Technology enabled more confidence in partnerships between adolescents and teachers (CPGM [20], SAPT [42]) - Users of Glucophone reported that most schools were supportive of use, although some reported objections [20] - Teachers and parents felt better equipped and confident with the responsibility of caring for a young person with T1DM when CGMs were used [49] |
| Data sharing between young people and parent | - Increased freedom and a safety net for adolescents (CGM [39, 49]) - Shift to independence using technology in adolescence – parents sometimes seen as intrusive (CGM [39], SAPT [42]) - Oscillated between being a source of conflict and resentment, and a sense of independence (CGM [39]) - Adolescents reported that using a Glucophone did not improve relationships with parents – they preferred that parents did not receive data in real-time [20] - Need for boundary-setting in relation to data sharing (CGM [39], SAPT [42]) |
| **5 Perceived impact on blood glucose control** | |
| Young people | - Steadier morning glucose levels (CGM [38, 40, 41, 51]) - Hypoglycemia prevention/ improved BG control (CGM and insulin pump [44]) - Stable BG levels (HCL [37], CL [48]) - Better management – less likely to over-correct lows/highs leading to other issues (CGM [38]) - Increase in percentage time in target range; steadier glucose levels (HCL [37]) - Overall no significant difference in glycemic control between users and non-users of CSII [45] |
| Parents | - Easier to achieve glucose targets (CGM [46]) - Enabled improved snacking and bolusing habits, regulating post-prandial BGLs (CGM [51]) |
| Both | - Reduced frequency and severity of hypoglycemia (CGM [47]) - Easier to achieve glucose targets (CGM [47]) - Lower HbA1c levels [20, 46] |
| **6. Device design and features** | |
| Quality | - Difficulty inserting and/or removing the device, and finding a comfortable and discrete and comfortable place on the body to locate it (CGM [38]) - Buttons falling off and issues with power port cover (CGM [41]) - Loud noise of insulin syringe holder button [40] - Lack of light or noises (discreet) but difficult to use at night (CGM [40]) |
| Equipment and size | - Speed and discreteness (insulin pump when compared with an insulin pen [40]) - Sensor, pump and transmitter too big (CL [48], CGM [52]) - Bulky [44], chunky [40], uncomfortable (HCL [37], CGMs [40, 49], insulin pumps [40]) - Finding a comfortable and discrete place to locate the device (CGM [38]) - Too much equipment was a constant reminder of living with T1DM (CGM [40, 49]) - Issues with having a second cannula available (CL [48]) - Sensors falling off (FGM [46], HCL [37]) - Tape adhesive issues (FGM [46], SAPT [42], CGM [44, 51]) - Environmental concerns related to single use only resources (disposable insulin pens [40]) |
| Data trends | - Useful in understanding BGL trends (CGM [38, 40, 49, 51], CPGM [20]) - Parents found trends more useful than did adolescents (CL [48]) - Retrospective analysis useful for informing decision-making (CGM [38, 51]) - Knowing how much insulin left in pump and matching bolus to food intake (CGM [40]) - Overwhelming amount of data (CGM [49]) |
| Data lag | - Lag between interstitial and blood glucose levels leading to data distrust (FGM [46], CGM [38, 51], SAPT [42]), and mistrust in device and own embodied experience [40] - However, for others mistrust in the device led them to gain trust in their embodied experience (SAPT [42]) |
| Connectivity and calibration | - Issues with connectivity to monitors, pumps or radio features (CGM [50], CL [48]) - Difficulty integrating SAPT with CGM [42] - Need for regular recalibration and related time-commitment frustrating (CGM [38, 51], SAPT [42], FGM [46], HCL [37], CL [48]) - Requires too much information related to meals, boluses and corrective insulin doses (HCL [37]) - Adolescents using CPGM reported problems with poor service to rural areas, or having to send the phone for repairs (74% of using Glucophone), yet in general the majority liked the phone/ meter combination (74% of youth and 85% of parents) [20]. |
| Discomfort related to devices | - Painful insertion (FGM [46], CGM [38, 49, 52], insulin pump [43], SAPT [42]) - Difficulty inserting device (CGM [38]) - Insertion site pain, irritation, or bruising (CGM [44], FGM [46]) - Sensitivity to tape (FGM [46], SAPT [42], CGM [44, 51]) - The need to rotate sites can lead to locations difficult for child to reach to insert cannula for pumps/MDI and need to rely on parents to assist (CL [43]), which was embarrassing for some adolescents (SAPT [42]) |
| **7. Cost** | |
|  | - Barriers to self-funding technologies were: cost of CGM and associated supplies [40, 49]; and short life-span of SAPT sensors [42] - If having to choose one due to cost, CGM was often opted for over an insulin pump due to the perception greater value of continuous data [49] - Financial barriers to the acquisition of CGMs for some had an emotional impact on some who felt grateful that they could afford them [49] - Adolescents worried about the high cost of SAPT and in relation to this, about losing or damaging them [42] - Main reason for ceasing use of FGM was the cost, FGM became reimbursable under the French National Health Insurance program on 1st June 2017 (at the end of the study) [46] - Changes to government subsidies for CGMs (often newly subsidised ones not as good as previous, or strips no longer subsidised, meaning forced to change to a new poorer quality product) [40] - CGM expensive for those who self-fund, not funded by NHS and concern about having to give it up if finances are tight [51] - If it was covered by insurance 80% parents said they would use Glucophone for child’s care [20] |
| **8. User satisfaction** | |
|  | - Majority of young people and parents satisfied (FGM [46], CGM [50]) - Overall high satisfaction with FGM use in 1-18year olds. Most frequent reason for dissatisfaction with FGM was the absence of real-time alerts [46] - Higher satisfaction scores for those who use CGM > 6 days per week when compared with < 4 days per week. Parents reported higher satisfaction scores than adolescents (CGM [52]) - No statistically significant difference in attitudes and experiences between adolescent users and non-users of CSII who reported overall positive attitudes towards self-management [45] - Overall, users of closed loop technology were satisfied, least favourable rating for both adults & children related to “size, weight, appearance” and “use during sports, exercise and bathing” [48] |
